# On the pitfalls of peer review

**DOI:** 10.12688/f1000research.7342.1

**Published:** 2015-11-11

**Authors:** Wilfred van Gunsteren

**Affiliations:** 1 Laboratory of Physical Chemistry, Swiss Federal Institute of Technology (ETH), Zürich, CH-8093, Switzerland

**Keywords:** research manuscripts, research proposals, faculty appointment, faculty tenure, peers, performance indicators, institutional review, editorial process

## Abstract

The review process of academic, scientific research and its basic tenets is considered, thereby distinguishing between (i) reviewing of manuscripts to be published in the scientific literature, (ii) reviewing of research proposals to be financed by funding agencies, (iii) reviewing of educational or research institutions with respect to their proper functioning, and (iv) reviewing of scientists with the aim of appointing or tenuring faculty.

## Introduction

Scientific research is primarily driven by curiosity, by the desire to determine relationships between observations and to develop models that can be used to understand and predict phenomena in the world in which we live. It is a continuous process of refinement and extension of knowledge and understanding. Every generation of researchers stands on the shoulders of those who have gone before. This is why reporting research results, be it positive or negative ones, in the scientific literature is of importance to the progress of our understanding and knowledge. The scientific literature constitutes the database of scientific knowledge. Because any research may be flawed due to erroneous observations, overseen correlations, incorrect assumptions, or just sloppy reasoning, any model or theory that is proposed must be justified and tested against data that are both tangible and publicly available. Publication of research results allows other scientists to check the validity of a proposed model or theory without having to repeat the original work, thereby facilitating scientific progress. Thus the integrity of published research is of fundamental value to the academic community of scholars.

Since the 18th century quality control of research publications has been exerted by peer review: the judgement of scientific reports by academics with equivalent knowledge. Peer review can only function under the umbrella of the ethics of science ^1^. This assumes an unbiased examination of the opinion or data based on logical and empirical criteria and places trust in the competence and honesty of the reviewers, be they a colleague or a competitor. However, with the expansion in both the size and number of research institutions over the past half century, the number of research publications has grown rapidly, reaching about 1.4 million per year in 2013. To properly evaluate such a large number of manuscripts is a challenge and puts severe strain on the peer review system. The increasing mass of submitted manuscripts of decreasing quality and relevance is slowly choking the review system and thus slowly corrupting the database of knowledge ^2^. It also leads editors of journals to use odd arguments, “I regret to inform you that our editors have now carefully considered your manuscript and feel it unsuitable for publication as we have not been able to secure reviews.” A *testimonium paupertatis*.

Peer review is also called upon by agencies funding research in the process of evaluating research proposals in regard to funding. This role of peer review adds a financial dimension to the review process: money that is allotted to the research of a competitor will generally not be spent on research of the reviewer. One only has to remind oneself of the steady stream of scandals in the banking industry to fear the impact of money upon the ethics of science.

A third type of reviewing is the evaluation of educational or research institutions such as universities in regard to how they function compared to that envisaged by the boards of such institutions. This role of peer review adds yet further dimensions to the review process including the goals and outcomes in teaching of science and research, and the effectiveness of the corresponding organisational structure. Here peer review requires more than reading and judging manuscripts and research proposals.

Peer review is also invoked by universities when recruiting faculty. Apart from the ability to offer inspiring teaching and original and high-quality research, aspects of a candidate’s personality are to be gauged: openness of mind, views on academic ethics, sensitivity to academic issues broader than research and teaching, for example. Here peer review requires ‘seeing through’ a person with respect to psychological, social, ethical and organisational abilities.

## Review of manuscripts to be published in the scientific literature

In view of the development of the internet and its storage capacity, one could consider the option to refrain from peer review and allow anyone to publish whatever she or he wishes. As the development of Wikipedia has shown, absence of any form of review may quickly lead to a loss of reliability of the material published. Thus some validation ^3^ and selection of submitted manuscripts by peer review seems necessary to avoid too much corruption of the database of scientific knowledge. A reviewer should formulate an opinion on the quality of a manuscript:

1. Clarity of text, tables and figures.
2. Reproducibility of the results from the data specified.
3. Sound connection between the results and the conclusions (no overstatements).
4. Embedding of the results in the literature (proper referencing).
5. Relation to other methods addressing the same problem.
6. Novelty of the method or results.
7. Relevance of the results to the scientific community.

The first six aspects can be addressed objectively, whereas judgement of the relevance of particular research is subjective. Reviewers should not prescribe what they think to be important and should be written or omitted from the manuscript, or prescribe particular references remotely related to the topic to be included ^4, 5^.

The quality of the reviewer’s report is to be evaluated by the editor who requested it:

1. Apparent knowledge of a reviewer regarding the subject of the manuscript.
2. Validity and consistency of the arguments of a reviewer. I have seen reviewers who initially judge the reported research to be “relevant, a novel approach and publishable”, but after the authors had refused to comply with an inappropriate request by the reviewer, then judge the manuscript as “not providing physical insight and inappropriate for publication in the journal”.
3. Possible bias because of a vested interest of a reviewer, e.g. reflected in criticism of an Introduction or Discussion, without criticism of the results themselves.

Editorial decisions should be consistent as function of time. I have seen an editor asking for the addition of data and, upon this request being honoured, rejecting the manuscript.

One way to avoid your work being reviewed and edited by persons with insufficient knowledge of the field is to submit to journals maintained by professional organisations such as the national chemical or physical societies. These suffer less from sensationalism and are less influenced by hypes when selecting manuscripts. As a colleague once confided when questioned about overstating his results: “Of course you tone down the wording of a manuscript after it has been accepted for publication by Science or Nature.” Or, as a former colleague at the ETH once said: “Why lose time arguing with incompetent reviewers or editors of a high-profile journal, if you can get your work competently reviewed and smoothly published in a quality journal such as Helvetica Chimica Acta? If the published science is of real, lasting importance, it will sooner or later be noticed, irrespective of the journal.”. A genuinely academic opinion.

## Review of research proposals

Funding agencies also use peer review to select research proposals for funding ^6, 7^. Often particular research goals are set, such as relevance to society or to the development of methods, tools or materials of practical interest, e.g. for industry. Innovation is a much cherished, frequent request. This leads to scientists echoing these goals in the introduction of the proposal, e.g. claiming they will develop multiple drugs to treat wide-spread diseases such as AIDS, stroke or dementia, while the proposed research itself is at best only remotely related to achieving this goal. Such a discrepancy between claims and content in a proposal is at odds with the ethics of science and undermines the credibility of the scientist and as a result the chance of getting the proposal approved, because the credibility of the researcher must be considered by the reviewer when answering the question as to whether the proponent will be able to successfully carry through the proposed research. Obtaining funding for basic science and risky but well-thought-through projects with a long-term perspective becomes difficult if short-term relevance is requested by agencies ^8^.

Research proposals should be judged in terms of (i) the attainability of the stated goals using the proposed means, (ii) the risk of failure versus the resources requested, and (iii) the ability of the proponent to carry through the proposed research. These must be considered while always remembering that the result of an exploration of uncharted territory cannot be planned, no matter how many milestones are requested. One can plan to put a man on the moon, but not to invent a new material.

## Review of educational research institutions

Universities regularly use peer review to evaluate the performance of their different departments regarding three major aspects of their activity: (i) effectivity and content of their teaching, (ii) quality and novelty of their research, and (iii) effectivity of their organisational structure. Such peer review also has its pitfalls. Not only are more aspects involved than only the quality of research, but also the sheer number of scientists and personnel to be evaluated makes this a nearly impossible task to execute based on a site visit lasting just a few days. Of course one could require the reviewing committee to spend more time at the institution, but this will reduce the willingness of good scientists to participate in reviews. I was once asked to chair a committee tasked with evaluating the performance of the chemistry departments of ten Dutch universities. The agency in charge estimated the time required would be 45 days. When I asked whether they thought the president of the ETH would appreciate me spending about nine weeks in The Netherlands, I did not get an answer from the agency. It is also almost impossible to obtain a reliable impression regarding the teaching abilities of staff during a visit of a few days. In addition, increased specialisation hampers institutional review: it is nearly impossible to cover all types of research performed in a large department by a review committee consisting of even 10 to 15 scientists. Most have much less.

The effectivity of an organisational structure can only be judged by reviewers who are familiar with the socio-cultural and political environment of the institution. For example, an organisational structure that functions well within the context of the US culture and research landscape based on funding through research agencies and foundations may be not appropriate in a European context where funding is primarily through government channels. Or, teaching to British high-school graduates may require an approach different from teaching to graduates from German or Swiss Gymnasiums.

In view of these odds, and because an academic institution generally changes rather slowly, it would be wise to limit institutional review to once in say 10 years, and to select a reviewing committee consisting of scientific peers with academic experience and a sense for the socio-cultural environment of the institution to be reviewed.

## Review of scientists with respect to appointment or tenure

One of the most important tasks of university management is recruiting of faculty. Any error made will have lasting detrimental effects due to the long residence times of faculty and its central role in teaching and research and when serving as peers. The procedure of nomination of faculty and the role of peers in it should strike a balance between the opinion of scientific peers in a selection committee who are knowledgeable in the particular field of research of an open faculty position and who may judge the quality and originality of the research of a candidate on the one hand, and the opinion of the other members of the committee who are knowledgeable in other fields of research on the other hand. The latter may judge clarity of presentation and the maturity of the personality of a candidate without possibly being biased by feelings of collegiality with persons working in their field.

At the ETH this is secured by a selection committee composed by the president of the ETH upon proposal by the department in which the faculty position is located, and in which members from other departments and from outside the ETH constitute a majority, with an independent chairman of the committee, chosen from a pool of such chairs, and a secretary from the staff of the president ^9^. Since the peers that are members of the department are a minority, they must convince the outsider peers of the quality of a candidate, a barrier against co-optation within a field of research. Yet the majority will follow the minority of department members in two cases. If the department members of the committee express their minority wish (i) to invite a particular candidate for a research presentation and interview, and (ii) to veto a candidate favoured for the faculty position by the majority of the committee, because they expect not to be able to work with the selected person. For a committee of 10 to 15 peers from different departments and institutions, judgement of research and teaching abilities of candidates should not be too difficult. But, for a thorough evaluation of personality characteristics a single research presentation and an interview by the committee may not be sufficient. One would rather observe how a future colleague would function in the different roles of a faculty member of a university. The latter is much easier when evaluating candidates for tenure. The members of the department of a tenure candidate have the opportunity to observe the functioning of a tenure-track professor a few years before proposing tenure to the tenure committee, which then only has the duty to see to it that the quality standards in research and teaching are fulfilled.

Essential for recruiting of faculty is the composition of a selection committee in regard to judging research, teaching and personality of candidates and its ability to conduct an open and honest discussion on real issues regarding candidates, i.e. not on scientifically rather irrelevant issues ^10^ such as citations of recent research publications, i.e. short-term popularity, h-indices or grant money gathered. Being one of many co-authors of a paper in a high-profile journal such as Science or Nature is not to be considered to reflect scientific quality or long-time vision.

## The use of indicators of performance

The time pressure on reviewers will inevitably induce them to rely on performance indicators rather than spending time to investigate in depth the research of a scientist. However, measurement results in numbers, and numbers reflect quantity, not quality. Quality cannot be caught in a number. It is also seductive to compare numbers ^10– 12^. In other words, numbers lead to rankings, and rankings lead to competition. Excessive competition undermines care and rigour, encouraging activities close to or, ultimately, beyond the boundaries set by the ethics of science ^2^. The increasing pressure to violate academic principles is illustrated by the mounting number of cases of plagiarism and scientific fraud ^13^. Focus on quantity as opposed to quality also leads to the aversion of risk: truly difficult and innovative research is shunned. A focus on competition will not enhance the quality of research. Quality measured by metrics alone is an illusion and the cost to society is growing inefficiency ^14^.

Indicators such as number of citations of publications, grant money gathered, number of successful students educated, or student satisfaction are only useful to detect extremes. A curriculum vitae with more than 1000 research publications must raise questions regarding the true involvement of the person in question in the research and the scope of the issues addressed. On the other hand, a lack of publication activity may indicate a lack of effort, the inability to finalise work, or reflect the difficulty of the research being executed. Student evaluations of courses are dependent on the difficulty of the topic, whether the course was logically structured, on the size of the class, whether the course was obligatory, how many credit points could be earned, the knowledge of the students, etc.. As a teacher I consistently received higher marks for an optional 3rd year course on algorithms and programming, a well-structured topic taught to a class of about 20, than for a general obligatory freshman course on computer science for about 200 chemists and biologists, for whom the topic was not their primary interest. High marks for teaching may reflect more the ability to entertain than to inspire and teach. Low marks may reflect a lack of interest by the students in the subject as much as genuine lack of clarity.

One of the most perverse consequences of the growing importance of rankings and competition between universities I have seen was a quarter-page advertisement in a daily newspaper in The Netherlands. In it, the University of Utrecht thanked five of its scientists for having obtained a European Research Council Consolidator Grant. My parents – although not being graduates of this university - would turn in their grave, they used to say “Science needs no applause”, an echo from times gone by?

## What’s to be done?

Science lives from an open exchange of arguments and data. It is damaged by reviewers prescribing what kinds of arguments can or cannot be published. Research proposals should not be evaluated by reviewers easily impressed by hype or unjustified promise of utility in order that scientists who follow trends, promising much but delivering poorly, can be barred from funding. The larger the gap between proposals, publications and scientific reality, the greater the long-term damage to the academic community of scholars and its credibility will become. If the curriculum vitae of an applicant for a faculty position lists the number of citations or an h-index value or the amount of grant money gathered, this is to be regarded as a sign of superficiality and misunderstanding of the academic research endeavour, a basic flaw in academic attitude, or at best as a sign of bad taste ^2^.

To maintain their credibility as impartial peers, reviewers should refrain from reviewing work by close collaborators and should be conscious of potential bias when reviewing work of colleagues. Networks of scientific friends that review the work of each other in an unjust manner undermine scientific integrity. They constitute a perversion of the ethics of science ^5^. Yet, excluding from review all persons with whom a proponent of a research proposal has a joint publication may lead to equally perverse outcomes. As a former vice-president for research of the ETH discovered, this rule excluded more than 260 scientists familiar with a given field from reviewing an ETH research grant proposal.

The process of review by peers has its pitfalls. It needs to be handled with care and a sense of proportion. There is, however, no viable alternative. Using indicators, which primarily reflect quantity, not quality, leads to perverse incentives and should be avoided ^14^.
