# Supplementary material for: On the pitfalls of peer review
Source: F1000Res. 2015 Nov 11;4:1244. [Version 1] doi: 10.12688/f1000research.7342.1 (PMC4669999; doi:10.12688/f1000research.7342.1)
Supplement: Peer review history

# Peer review: Referee response for version 1

**DOI:** 10.5256/f1000research.7912.r11208

**Published:** 2015-12-03

**Authors:** Daan Geerke

**Affiliations:** 1 AIMMS Division of Molecular Toxicology, Department of Chemistry and Pharmaceutical Sciences, Faculty of Sciences, VU University Amsterdam, Amsterdam, Netherlands

Starting from a systematic overview of the reviewing process, the current paper summarizes the true values and the possible pitfalls in scientific and academic refereeing. It can also be seen as a (very) useful guide for anyone involved in reviewing, including those who are making policy for the assessment of individual scientists, faculties, research schools and institutes, which is increasingly putting additional pressure to academic staff. The striking examples given by the author illustrate the strong need to keep (or even start?) discussing the mode of reviewing within scientific communities. In line with the previous referee report, I can also exemplify this need by personal experiences with peer reviewers judging the quality of grant applicants based on the number of papers in journals with impact factor > 10, or with universities bringing out press releases about their top researchers defined as grant awardees. Therefore the current paper is in my opinion not only a very good but clearly also a highly timely overview of pitfalls in peer reviewing.

I have read this submission. I believe that I have an appropriate level of expertise to confirm that it is of an acceptable scientific standard.

# Peer review: Referee response for version 1

**DOI:** 10.5256/f1000research.7912.r11210

**Published:** 2015-12-01

**Authors:** Xavier Daura

**Affiliations:** 1 Institute of Biotechnology and Biomedicine, Universitat Autònoma de Barcelona and Catalan Institution for Research and Advanced Studies (ICREA), Barcelona, Spain

Peer reviewing an article on the pitfalls of peer review reminds you of the responsibility of an act that, due to time pressure, we often do less proficiently than we should. In addition, if the author of the manuscript happens to be a friend, you get directly confronted with the essence of the topic. Can we be impartial about the work of friends and direct competitors? My own opinion is that we ought to if we want to be credible as a collective; but we need to be transparent about it. In more practical terms, as van Gunsteren puts it, finding peers in the same field of expertise both with no connection and in no competition with a given author could be an arduous task. If we talk about an opinion article it is even questionable that the reviewer should prescribe the author any changes (assuming the article is void of nonsense, excess of commonplace arguments or bad writing). Opinion articles are probably the most personal form of scientific writing, their only requisites being the ability to provoke thought and generate discussion on a topic of interest. The article by van Gunsteren fully complies with these principles. It also has a wider focus than recent articles on peer review, thus including the evaluation of publications, projects (ex-ante, but a similar discussion would be valid for ex-post evaluations), institutions and researchers. The discussion of the four levels of peer review is illustrated with examples, understandably centred on the author’s own experience. Some of the examples would seem anecdotal wouldn’t we all have similar experiences to tell. In summary, a concise, easy-reading article worth adding to recent discussions on an evaluation system that while far from ideal is still the best we have.

I have read this submission. I believe that I have an appropriate level of expertise to confirm that it is of an acceptable scientific standard.
